# Annual Report of the Health Physician of the City of Buffalo, for the Year 1855

**Published:** 1856-05

**Authors:** 


*Annual Report of the Health Physician of the City of Buffalo, for the year* 1855. By John Root, M. D., Health Physician.

Dr. Root makes a brief and unpretending report of the facts of his term of office. During tha year no epidemic occurred, and sporadic diseases were far less fatal than usual. The preceding year was an epidemic one, but is the only one with which any fair comparison can be made.

The total number of deaths in the city

| | | |
|---|---|---|
| For the year 1854, was, - - - - | | 2936 |
| For the year 1855, " - - - - - | | 1856 |
| Decrease in mortality, - - - | | 1080 |
| In 1854 the deaths from cholera were - | 512 | |
| " 1855 " " " - - | 11 | |
| Decrease in mortality by cholera, - - | | 561 |
| Decrease in the mortality of other diseases than cholera, | | 519 |

The rate of mortality for the year was only one in $43\frac{10}{100}$ of the entire population, being considerably above the average of the most healthy cities.

At the small-pox hospital there were, during the year, only nine patients, against fifty-eight during 1854.

| | | | | | |
|---|---|---|---|---|---|
| Of the total number of deaths there were | | | | Americans, | 775 |
| " | " | " | " | Foreigners, | 904 |
| " | " | " | " | Unknown, | 177 |
| " | " | " | " | Males, | 1066 |
| " | " | " | " | Females, | 790 |
| " | " | " under 5 years of age there were | | | 1085 |

being about $\frac{9}{15}$ of the whole mortality. This is a frightful comment on the value of infant life in cities.

Dr. Root will accept our thanks for his Report, as well as for the prompt manner in which he has furnished us the monthly reports during the year.

